# A Meta-Analysis of the Relationship between Cigarette Smoking and Incidence of Myelodysplastic Syndromes

**DOI:** 10.1371/journal.pone.0067537

**Published:** 2013-06-21

**Authors:** Hongyan Tong, Chao Hu, Xiufeng Yin, Mengxia Yu, Jun Yang, Jie Jin

**Affiliations:** 1 Department of Hematology, The First Affiliated Hospital of Zhejiang University, Hangzhou, People’s Republic of China; 2 Institute of Hematology, Zhejiang University School of Medicine, Hangzhou, People's Republic of China; 3 Department of Hematology, The First Affiliated Hospital of Ningbo University, Ningbo, People's Republic of China; 4 State Key Laboratory for Diagnosis and Treatment of Infectious Diseases, The First Affiliated Hospital of Zhejiang University, Hangzhou, People’s Republic of China; West Virginia University School of Medicine, United States of America

## Abstract

**Background:**

In recent years, epidemiologic studies have reported controversial results relating cigarette smoking to myelodysplastic syndromes (MDS) risk. A meta-analysis was performed to assess such potential relationship between cigarette smoking and incidence of MDS.

**Methods:**

A search of literature published before October 2012 for observational studies evaluating the association between cigarette smoking and MDS, returned 123 articles and of these, 14 were selected for this study. The outcomes from these studies were calculated and reported as odds ratios (OR). Quality assessments were performed with the Newcastle-Ottawa Scale. Heterogeneity was evaluated by the I^2^ index and source of heterogeneity was detected by sensitivity analyses. Finally, publication bias was assessed through visual inspection of funnel plots and Egger’s test.

**Results:**

The pooled OR of developing MDS in ever-smokers was 1.45 (95% CI, 1.25 to 1.68) versus non-smokers. Current and former smokers had increased risks of MDS, with ORs of 1.81 (95% CI, 1.24 to 2.66) and 1.67 (95% CI, 1.42 to 1.96), respectively. In subset analyses, ever-smokers had increased risks of developing MDS if they were living in the United States, or in Europe, female in gender, had refractory anemia (RA)/RA with ringed sideroblasts (RARS) or RA with excess blasts (RAEB)/RAEB in transformation (RAEBt), respectively. Our results demonstrated that the association was stronger in individuals who smoked ≥20 cigarettes/day (OR, 1.62; 95% CI, 1.03 to 2.55) versus those who smoked <20 cigarettes/day (OR, 1.36; 95% CI, 1.13 to 1.64). Moreover, individuals who smoked more than 20 pack-years had increased MDS risk (OR, 1.94; 95% CI, 1.29 to 2.92).

**Conclusion:**

Our outcomes show that smoking increases the risk of developing MDS in ever-smokers who are current or former smokers. We also demonstrate here that positive association between cigarette smoking and risk of MDS exists, and occurs in a dose-dependent manner.

## Introduction

Myelodysplastic syndromes (MDS) are a heterogeneous group of neoplastic clonal stem cell malignancies that present clinically as anemia, thrombocytopenia, leucopenia, and ineffective bone marrow hematopoiesis. Patients who are diagnosed have substantial risk for transformation into acute myeloid leukemia (AML) (10–40%) [1]. Diagnosis can be categorized into subtypes according to histological, genetic characteristics and immunological. Historically, though, MDS has been made using the French–American–British (FAB) classification, with subtypes being refractory anemia (RA), RA with ringed sideroblasts (RARS), RA with excess of blasts (RAEB), RAEB in transformation (RAEB-T), and chronic myelomonocytic leukemia (CMML)[2].

At the present time, the most widely used system for risk stratifying MDS patients is still the International Prognostic Scoring System (IPSS)[3]. The IPSS incorporates three factors: (1) the percent blasts in the bone marrow, (2) the number of peripheral cytopenias, and (3) the karyotype. According to these factors, a score is calculated which results in placement into an IPSS Risk Group (Low, Intermediate-1, Intermediate-2, or High Risk). Despite advances in new therapeutic methods, MDS remain incurable. Patients with MDS, especially those with high-risk MDS, have an adverse prognosis. Therefore, a better understanding of the etiology of this disease may lead to significant reduction in MDS incidence.

Although a wide variety of factors have been studied for their connection with cancers, few are considered risk factors for the development of MDS. For humans, smoking is a well-established carcinogenic factor, with the most recent monograph by the International Agency for Research on Cancer (IARC) listing cancer of the lung, oral cavity, larynx, pharynx, stomach, uterine cervix, liver and myeloid leukemia as being causally linked to smoking [4].

Some studies of MDS have reported a dose-response effect according to the duration and/or intensity of smoking, while others showed no such effect. Identifying a relationship between cigarette smoking and MDS would make smoking cessation an appealing measure for prevention of MDS. Thus, we are interested in further investigating cigarette smoking and the development of MDS and conduct here a meta-analysis of published literature to investigate whether an epidemiologic relationship, if any, exists between the risk of MDS and cigarette smoking.

## Materials and Methods

### Literature Search

Systematic literature search was conducted by two independent reviewers (Chao Hu and Mengxia Yu) in PubMed, the Cochrane Library and Embase database for papers published before October 2012. The following search terms were used: (myelodysplastic syndrome OR MDS OR myelodysplastic OR myelodysplasia OR preleukemia) AND (smoking OR tobacco OR cigarette). The titles and abstracts of the resulted articles were checked. After excluding nonrelated articles, full-text articles were retrieved. References of related articles and reviews were checked for additional articles.

### Inclusion and Exclusion Criteria

Eligible articles should meet all the following criteria: (1) studies were case-control or cohort studies; (2) studies assessed the association between cigarette smoking and the risk of MDS; (3) odds ratio (OR) estimates and their 95% confidence intervals (95% CI) were reported or could be calculated; (4) the identified studies were reported in English. Any discrepancies between reviewers on inclusion of a study were resolved by joint evaluation of the manuscript. In the event of multiple publications from the same study or overlapping study populations, only the most relevant one was selected. Reviews or editorials, letters to the editor without original data and case reports were excluded.

### Data extraction

Data extraction included first author’s name, year of publication, country of origin, study period, study design, gender, age, sample size (cases and controls or cohort size), method of ascertainment of smoking, criteria for diagnosis of MDS, the outcome measured with 95% CIs, matching and adjusted covariates. If the required data for the meta-analysis were not available in the published article, we made contact with the corresponding authors for missing data. In the event of disagreement between the two reviewers, a third reviewer extracted the data and results were attained by consensus. Considering the rare incidence of MDS, the relative risk in prospective cohort studies was approximately the same as the odds ratio (OR) [5], thus, permitting the combination of cohort and case-control studies. Crude OR (unadjusted) and adjusted OR were all used for meta-analysis. The quality of each study was evaluated independently by two authors who used the nine-score Newcastle-Ottawa Scale (NOS) [6].

### Statistical analysis

Fixed-effect or random-effect models (the DerSimonian and Laird method) [7] were appropriately used to calculate a pooled OR with 95% CI. Heterogeneity was assessed by using Q-test and I^2^ index. P >0.05 for the Q-test indicated a lack of heterogeneity among the studies. The pooled OR estimate of each study was calculated by the fixed-effect model. Otherwise, the random-effect model was used. Sensitivity analysis was performed by sequential omission of individual studies under various contrasts to reflect the influence of the individual data to the pooled ORs and evaluate the stability of the results. Subset analyses were performed and categorized by geographical regions, disease subtype, sex, quantity of cigarettes smoked per day, years of smoking, and pack-years. An estimation of potential publication bias was executed by the funnel plot, in which the standard error of log (OR) of each study was plotted against its log (OR). An asymmetrical plot suggested a possible publication bias. Funnel plot asymmetry was evaluated by the method of Egger’s linear regression test, a linear regression approach to measure funnel plot asymmetry on the natural logarithm scale of the OR [8]. The significance of the intercept was determined by the t test suggested by Egger (P<0.05 was considered as the presence of statistically significant publication bias). The STATA 11.0 statistical software (Stata Corporation, College Station, Texas) was used for all the statistical analyses. P<0.05 was considered statistically significant.

## Results

### Search Results

**Figure 1:**
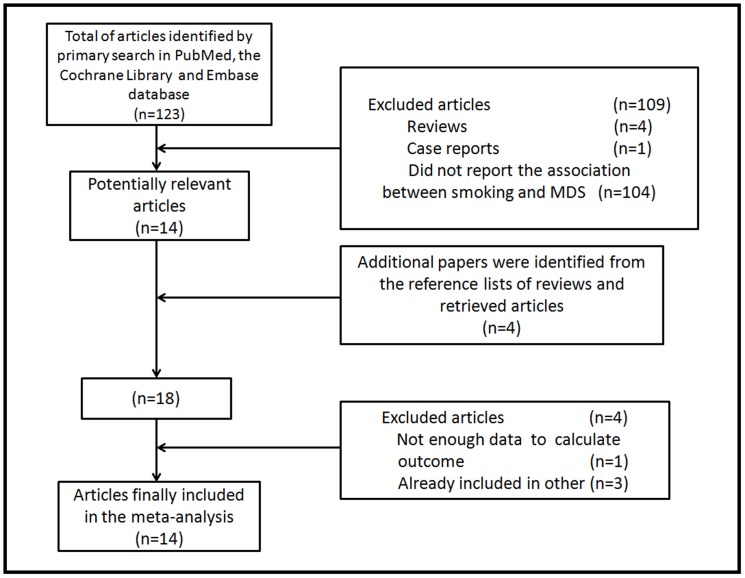
Flowchart of study selection process.

A total of 14 articles were selected for our meta-analysis, including one prospective cohort [9] and 13 case-control studies [10]–[22]. Our search flow was shown in Figure 1.

### Study Characteristics

The main characteristics of the included studies were provided in Table 1. Studies were published between 1991 and 2011. Three studies originated from the United States [9], [13], [22] and three studies from Asia [10], [17], [19]. The remaining eight studies were from European countries, including: two from Sweden [11], [16], two from Italy [18], [21], one from Greece [14], one from France [15], one from Serbia Montenegro [12], and one from UK [20]. A total of 2,588 MDS patients were included in this meta-analysis. Five studies reported a positive association between incidence of MDS and smoking [9], [13], [15], [18], [19]. For all studies in this meta-analysis, MDS was diagnosed by the FAB (French-American-British) or WHO (World Health Organization) criteria. Smoking habits were ascertained by personal interviews in nine studies [10]–[12], [14], [15], [18]–[21] and telephone interviews or mailed questionnaires in five [9], [13], [16], [17], [22].

**Table 1:** Main characteristics of cohort and case-control studies evaluating the association between cigarettes smoking and MDS.

					Case			Control					
Study	Country	Study Period	Study Design	Male	Age	Number	Male	Age	Number	MDS Diagnostic	Smoking	Study	Matching and Adjustments
				(%)	(years)		(%)	(years)		Criteria	Assessment	Quality	
Lv	China	2003–2006	Hospital-based	56.3	20–86	403	56.3	24–88	806	WHO	Face-to-face	6	Age, sex, anti-tb drugs, D860, traditional Chinese
(2011)^10^			Case-control								interview		medicine, alcohol intake, benzene, pesticides,
			study										gasoline, glues, hair dye, education, new building
Bjork	Sweden	2001–2004	Population-based	54.6	57–85	75	60.6	47–86	132	FAB	Interview	7	Age, sex, country of residence and area of living
(2009) ^11^			Case-control study								not specified		
Ma	United States	1995–2003	Cohort	71.0	57–78	193	NR	50–71	471799	FAB	Mailed	8	Age, sex, race, education, total energy intake
(2009)^9^											questionnaire		
Pekmezovic	Serbia	2000–2003	Hospital-based	51.3	20–85	80	51.3	NR	160	FAB	Interview	6	Age, sex
(2006)^12^	Montenegro		Case-control study								not specified		
Strom	United States	1999–2003	Hospital-based	69.4	24–89	352	64.8	25–89	443	FAB	Mailed	7	Age, sex, ethnicity, education, family history of
(2005) ^13^			Case-control study								questionnaire		hematopoietic cancer, alcohol intake, fertilizer,
													herbicide, pesticide, benzene, solvent, gasoline
Dalamaga	Greece	1995–2000	Hospital-based	55.9	44–85	84	55.9	47–85	84	FAB	Interview	6	Age, sex, marital status, education, alcohol
(2002) ^14^			Case-control study								not specified		consumption, time since first diagnosis of an
													autoimmune disorder
Nisse	France	1991–1996	Population-based	61.8	62–74	204	61.8	62–75	204	FAB	Face-to-face	8	Age, sex, oil use, agricultural workers, textile
(2001) ^15^			Case-control study								interview		operators, health professionals, living next to an
													industrial plant, commercial and technical sales
													representatives, machine operators
Bjork	Sweden	1995–1997	Population-based	60.6	52–83	326	61.1	50–82	333	FAB	Telephone	8	Age, sex, country of living, exposure to benzene,
(2000)^16^			Case-control study								interview		personal hair dye use
Nagata	Japan	1995–1996	Population-based	62.2	20–74	111	55.8	20–74	815	FAB	Telephone interview	8	Age, sex, living area
(1999)^17^			Case-control study								or mailed		
											questionnaire		
Pasqualetti	Italy	NR	Hospital-based	NR	16–91	85	NR	NR	85	FAB	Interview	6	Age, sex and institution
(1997)^18^			Case-control study								not specified		
Ido	Japan	1992–1993	Hospital-based	59.5	20–75	116	59.5	NR	116	FAB	Interview	6	Age, sex, hospital, hair dye use, occupational
(1996)^19^			Case-control study								not specified		exposure to organic solvents
West	UK	NR	Hospital-based	53.7	≥15	402	53.7	≥15	402	FAB	Interview	6	Age, sex, area of residence and hospital, year of
(1995)^20^			Case-control study								not specified		diagnosis
Mele	Italy	1986–1990	Hospital-based	64.9	≥15	111	34.4	≥15	1161	FAB	Face-to-face	6	Age, sex, education, residence outside study town
(1994)^21^			Case-control study								interview		
Crane	United States	1982–1984	Hospital-based	NR	≥18	46	NR	≥18	224	FAB	Telephone interview	6	Age, sex, alcohol intake, benzene, metal fume, dyes,
(1991)^22^			Case-control study								or mailed		glues, lacquers, varnishes, radiation, pesticides,
											questionnaire		paints, spray paints

NR: not reported ; tb: tuberculosis.

### Risk Estimation

**Figure 2:**
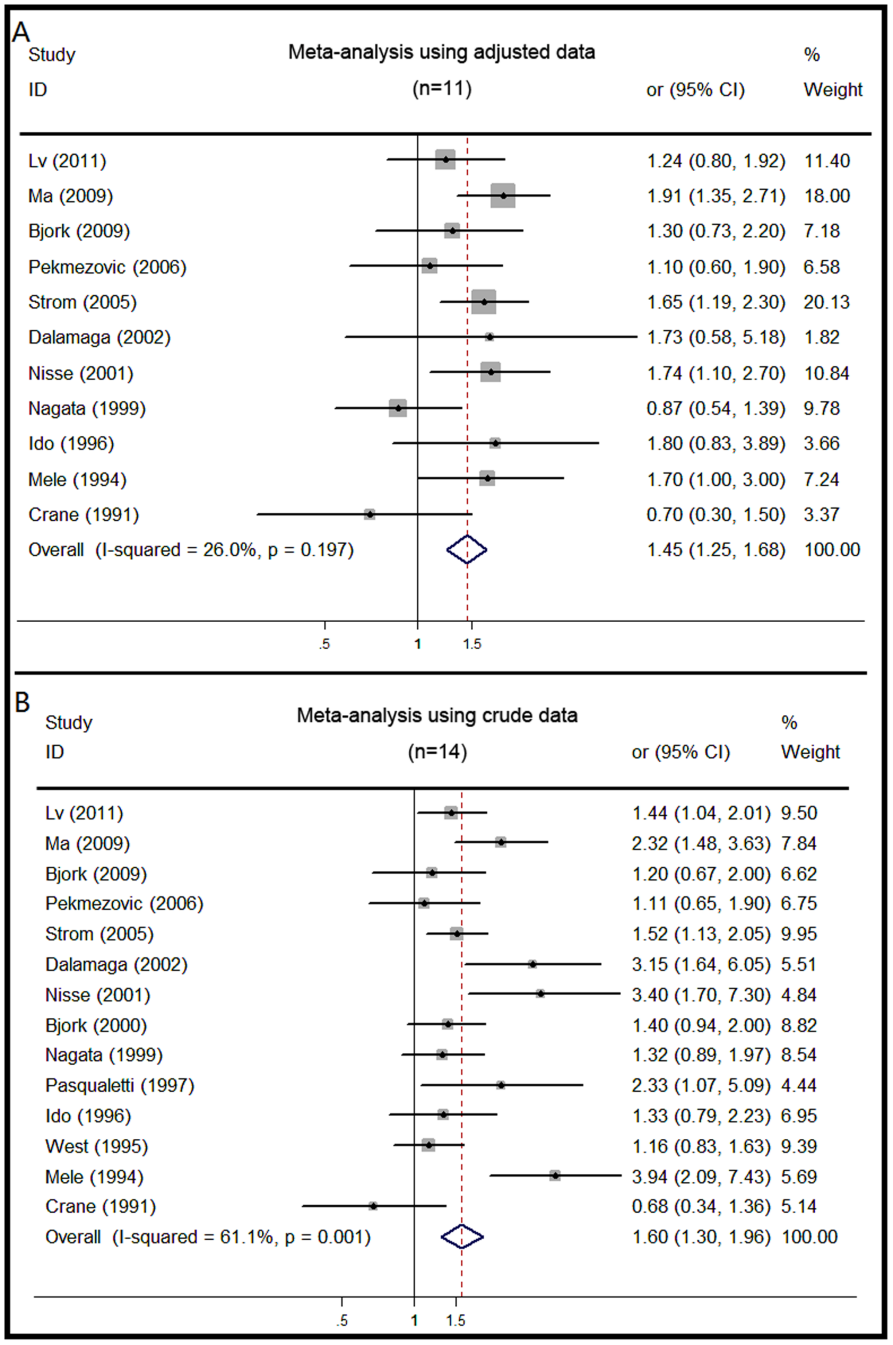
Forest plots showing risk estimates from cohort and case-control studies estimating the association between ever smoking and risk for MDS.

As shown in Figure 2, a significant association was seen between smoking (ever vs. never smoking) and incidence of MDS when using the adjusted data (OR, 1.45; 95% CI, 1.25 to 1.68), with moderate heterogeneity (I^2^ = 26.0%). The Egger’s test showed no evidence of publication bias (P = 0.273). The risk was similar for ever-smokers when using crude data (OR, 1.60; 95% CI, 1.30 to 1.96). Current smoking was also associated with increased risk for MDS (OR, 1.81; 95% CI, 1.24 to 2.66) (Figure S1A). There was moderate heterogeneity among studies (I^2^ = 60.5%), but publication bias was not found. An increased OR was discovered in former smoking (OR, 1.67; 95% CI, 1.42 to 1.96) (Figure S1B). The heterogeneity among studies was mild (I^2^ = 24.7%) and without publication bias (P = 0.895, Egger’s test). We executed sensitivity analyses and the result demonstrated that our study would not considerably affect the summary of risk estimates in ever-smokers, including current or former smokers.

### Subgroup Analyses

In table 2, we pooled the OR estimates by geographical region, gender, MDS subtype, smoking intensity, duration of smoking, and number of pack-years in ever smokers. The OR estimates showed cigarette smoking was consistently associated with an increased incidence of MDS when separately analyzed by each subgroup, although some of the results were not significant.

**Table 2:** Summary of pooled odds ratios of MDS for ever vs. never smoking in subgroups.

subgroup	Number of studies	Pooled OR (95%CI)	Q-test for heterogeneity P value (I2 score)	Egger’s test P value
Geographical region				
United States	3 (9, 13, 22)	1.84 (1.30, 2,06)	0.081 (60.3%)	0.217
Asia	3 (10, 17, 19)	1.14 (0.85, 1.53)	0.254 (27.0%)	0.542
Europe	8 (11, 12, 14, 15, 16, 18, 20, 21)	1.40 (1.18, 1.67)	0.648 (0%)	0.194
Sex				
Men	3 (13, 17, 19)	1.29 (0.95, 1.77)	0.879 (0%)	0.389
women	2 (13, 19)	2.02 (1.24, 3.31)	0.820 (0%)	-
MDS subtype				
RA/RARS	3 (13, 16, 19)	2.23 (1.50, 3.30)	0.889 (0%)	0.738
RAEB/RAEBt	4 (10, 13, 16, 21)	1.59 (1.21, 2.10)	0.771 (0%)	0.486
No. of cigarettes				
= 0	7 (9, 10, 11, 16, 17, 18, 20)	1	-	-
<20 and >0	6 (9, 10, 16, 17, 18, 20)	1.36 (1.13, 1.64)	0.278 (19.9%)	0.883
≥20	6 (9, 10, 11, 17, 18, 20)	1.62 (1.03, 2.55)	<0.01 (78.1%)	0.492
Duration				
= 0	5 (10, 11, 12, 16, 17)	1	-	-
<20 and >0	3 (10, 11, 17)	1.02 (0.66, 1.56)	0.533 (0%)	0.321
≥20	5 (10, 11, 12, 16, 17)	1.38 (0.90, 2.13)	0.011 (69.3%)	0.86
Pack-years				
= 0	5 (10, 11, 15, 16, 21)	1	-	-
<20 and >0	5 (10, 11, 15, 16, 21)	1.13 (0.88, 1.46)	0.216 (29.2%)	0.114
≥20	5 (10, 11, 15, 16, 21)	1.94 (1.29, 2.92)	0.038 (57.5%)	0.170

RA: refractory anemia; RARS: RA with ringed sideroblasts; RAEB: RA with excess blasts (RAEB); RAEBt: RAEB in transformation.

#### Geographical regions (Figure S2A)

An increased OR of ever-smokers was detected both in the United States (OR, 1.84; 95% CI, 1.30 to 2.06) and in Europe (OR, 1.40; 95% CI, 1.18 to 1.67), but not in Asia (OR, 1.14; 95% CI, 0.85 to 1.53).

#### Gender (Figure S2B)

When subgroup analysis was conducted by gender, a statistical significant adverse effect of smoking on developing MDS was observed in females (OR, 2.02; 95% CI, 1.24 to 3.31), but not in males (OR, 1.29; 95% CI, 0.95 to 1.77).

#### MDS subtype (Figure S2C)

Three studies reported data on RA/RARS [13], [16], [19]. Significant correlation was obtained (OR, 2.23; 95% CI, 1.50 to 3.30). Four studies reported data on RAEB/RAEBt [10], [13], [16], [21] and the results demonstrated that a significant association existed (OR, 1.59; 95% CI, 1.21 to 2.10). Both heterogeneity and publication bias were not observed in this sub-classification.

#### Smoking intensity

**Figure 3:**
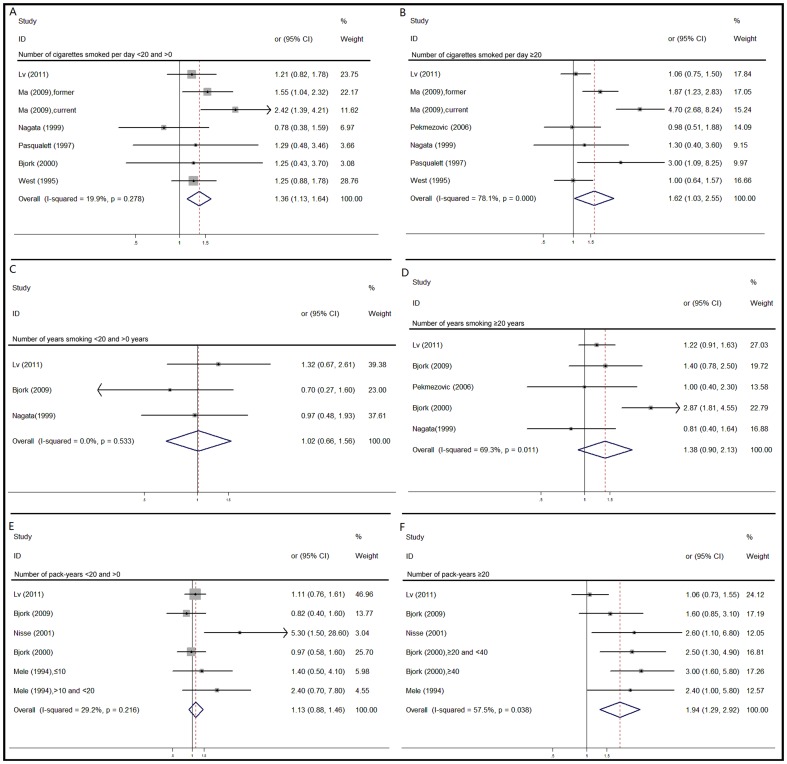
Forest plots describing the association between intensity, duration of smoking, number of pack-years and risk for MDS.

We used an empirical cutoff of 20 cigarettes per day to facilitate analysis. Ever-smokers who smoked fewer than 20 cigarettes per day, had an increased risk (OR, 1.36; 95% CI, 1.13 to 1.64) (Figure 3A). There was mild heterogeneity (I^2^ =  19.9%) and no evidence of publication bias. Ever-smokers who smoked more than 20 cigarettes per day, had a stronger association with development of MDS (OR, 1.62; 95% CI, 1.03 to 2.55) (Figure 3B). There was severe heterogeneity (I^2^ = 78.1%) without publication bias.

#### Duration of smoking

An empirical cutoff of 20 years of smoking duration was chosen. Among ever smokers who smoked for less than 20 years, there was no significant change to be observed (OR, 1.02; 95% CI, 0.66 to 1.56) (Figure 3C). Heterogeneity (I^2^ = 0%) and publish bias were not discovered as well. In individuals who smoked for more than 20 years, the OR was 1.38 (95% CI, 0.90 to 2.13) (Figure 3D) with moderate heterogeneity (I^2^ = 69.3%) without publish bias.

#### Number of pack-years

A cutoff of 20 pack-years was used to assist analysis. In individuals who smoked for fewer than 20 pack-years, the OR was 1.13 (95% CI, 0.88 to 1.46) (Figure 3E) with moderate heterogeneity (I^2^ = 29.2%) without publication bias. In individuals who smoked for more than 20 pack-years, a positive correlation was found (OR, 1.94; 95% CI, 1.29 to 2.92) (Figure 3F) with moderate heterogeneity (I^2^ = 57.5%) without publication bias.

## Discussion

Etiologic intervention represents an attractive, noninvasive approach of cancer prevention in at-risk individuals. The etiology of MDS is still not well understood and deserves ongoing investigation. Cigarette smoking is an established hazardous factor for cancer incidence and mortality [23]. However, prior literature has not provided a definitive link between cigarette smoking and risk of MDS. Therefore, we summarize here the current data available regarding this potential relationship and reveal several interesting points worth discussing.

Firstly, our study demonstrated a statistically significant association between ever-smokers and an increased incidence of MDS. Ever-smokers were shown to have a 45% higher risk of developing MDS than never-smokers, indicating that smoking played a vital role in the incidence of MDS. Further analysis revealed that current and former smokers had 81% and 67% higher risk of developing MDS than never smokers, suggesting that current smokers were under higher risk of MDS than former smokers. Same tendency had been reported in some previous epidemiological investigations [10], [13], [14]. One possible reason for the higher risk of current smokers was that current smokers might have a higher total cumulative dose and longer exposure time of smoking than former smokers. A recent study [13] demonstrated those who had quit smoking for longer than 15 years did not have excess risk of MDS. Such observation suggested that certain smoking-related damage may be reversible upon smoking cessation, but the effect of cessation may be only partially beneficial.

When we conducted subgroup analyses, our study illustrated that women who ever smoked had faced an added 102% risk of MDS. No such relationship was detected in men. Here, our results also indicated a stronger effect of smoking on RA/RARS than on RAEB/RAEBt (i.e., ever smokers had 123% increased risk of RA/RARS and 67% increased risk of RAEB/RAEBt, respectively). When subgroup analysis on geographical region was conducted, we noted that higher risk of MDS in United States (84%) than in Europe (40%), while no such association was observed in Asia. The different observations might be explained, at least in part, by the types of smoking (cigars, pipes, black or blond tobacco, oral snuff) across the world. In addition, a possible reason of racial differences should also be taken into account.

A direct relationship existed between higher numbers of cigarettes smoked per day/pack-years and increased risk of developing MDS. There was a high risk of MDS in individuals who smoked less than 20 cigarettes per day (41%). This risk increased to 79% in individuals who smoked more than 20 cigarettes per day. An insignificant risk of smoking on MDS was detected in individuals who smoked for less than 20 years, whereas a marginal association was detected if smoking continued for more than 20 years (OR, 1.38; 95% CI, 0.90, 2.13). When assessing the number of pack-years, smoking fewer than 20 pack-years did not show an increased risk of MDS. Nevertheless, smoking more than 20 pack-years increased the risk of MDS to 94%. Altogether, the data indicated not only the duration, but also that the intensity of smoking seemed to play crucial roles in the development of MDS. The positive relationship between cigarette smoking and incidence of MDS was thus dose-dependent.

The mechanisms by which cigarette smoking could affect the pathogenesis of MDS remain largely unknown. Several potential mechanisms, however, could support smoking as a risk factor for MDS. Generally, various chemicals contained in cigarettes, such as benzene, chromium, and formaldehyde, might contribute to the direct carcinogenicity. High level of benzene was reported among smokers [24], and chronic benzene exposure is known to cause bone marrow failure and lead to AML [25], [26]. Since MDS share many similar features of leukemia [27], it is possible that benzene might also increase risk of MDS. Indeed, one recent study confirmed that benzene was not only an independent risk factor of MDS, but also revealed a positive association between benzene exposure duration, level, and frequency and MDS development [10]. Hematology and immunological changes were also associated with cigarette smoking, such as affecting the number and activity of T cells, B cells, circulating natural killer cells, and macrophages [28]–[31] and reducing immunoglobulin production [32]. At the molecular level, smoking inhibited apoptosis by regulating Fas ligand and facilitated activation of nuclear factor-kappa B and other proinflammatory cytokines, like tumor necrosis factor α. These changes might, at least partially, attribute to the increased risk of developing MDS. In addition, some previous studies have demonstrated that smoking exposure might induce chromosomal defects in hematological malignancies. Reports have also shown smoking may be associated with specific MDS subtypes based on particular chromosomal signatures, while also influencing the prognostic impact of cytogenetic abnormalities on MDS survival [13], [16], [33], [34]. Smoking induced cytogenetic then alteration may be a potential mechanism for the increased risk of developing MDS. Further research, however, is needed to elucidate the underlying mechanisms.

The major strength of our meta-analysis was the collection of large studies of MDS to assess the relationship between cigarette smoking and MDS. This allowed us to explore in detail the association of interest among selected subsets using the meta-analytic method. However, as a meta-analysis of previously published observational studies, our study also had limitations affecting the interpretation of the results.

First, we did not uncover unpublished studies and chose to collect only published articles in English, which could bring publication bias, despite there being no significant evidence of publication bias observed in Egger’s test. Second, the ambiguous or varying definition of “smoking” in different questionnaires may have resulted in inaccurate estimates. Moreover, the recording of smoking habits in these questionnaires introduces a potential for recall or telescopic bias. Third, some of the subsets analyses, although specified a priori, were performed in small data set. Finally, methodological differences, as well as confounding factors and biases, inherent in cohort and case-control studies may have an influence on the results obtained by these studies.

In conclusion, results of this meta-analysis suggest a potential hazardous effect of smoking for developing MDS. The risk of MDS appears to be higher in women, in RA/RARS patients, and in heavy smokers. It is clear that smoking cessation has a positive impact on public health and should be advised globally. Based on our analyses, smoking cessation could also reduce the risk of developing MDS. Further study is warranted to confirm these findings and elucidate the likely biological mechanisms.

## Supporting Information

Figure S1 **Estimates of the odds ratio of developing MDS for (A) current smokers, and (B) former smokers.**
(DOC)

Figure S2 **Forest plots showing the odds ratio of developing MDS in different subgroups: (A) geographical region; (B) gender; (C) MDS subtype.**
(DOC)

Checklist S1 **PRISMA checklist.**
(DOC)

## References

[1] Greenberg PL, Young NS, Gattermann N (2002) Myelodysplastic syndromes. Hematology Am Soc Hematol Educ Program: 136–161. 10.1182/asheducation-2002.1.136 12446422

[2] Bennett JM, Catovsky D, Daniel MT, Flandrin G, Galton DA, et al (1982) Proposals for the classification of the myelodysplastic syndromes. Br J Haematol 51: 189–199. 6952920

[3] Greenberg P, Cox C, LeBeau MM, Fenaux P, Morel P, et al (1997) International scoring system for evaluating prognosis in myelodysplastic syndromes. Blood 89: 2079–2088. 9058730

[4] Group IW (2004) Tobacco smoke and involuntary smoking. IARC Monogr Eval Carcinog Risks Hum 83: 1–1438. 15285078 PMC4781536

[5] Zhang J, Yu KF (1998) What's the relative risk? A method of correcting the odds ratio in cohort studies of common outcomes. JAMA 280: 1690–1691. 9832001 10.1001/jama.280.19.1690

[6] Wells G, Shea B, O'Connell D (2009) Ottawa Hospital Research Institute: The Newcastle-Ottawa Scale (NOS) for assessing the quality of nonrandomised studies in meta-analyses. Available: http://www.ohri.ca/programs/clinical_epidemiology/oxford.asp.

[7] DerSimonian R, Laird N (1986) Meta-analysis in clinical trials. Control Clin Trials 7: 177–188. 3802833 10.1016/0197-2456(86)90046-2

[8] Egger M, Davey Smith G, Schneider M, Minder C (1997) Bias in meta-analysis detected by a simple, graphical test. BMJ 315: 629–634. 9310563 10.1136/bmj.315.7109.629 PMC2127453

[9] Ma X, Lim U, Park Y, Mayne ST, Wang R, et al (2009) Obesity, Lifestyle Factors, and Risk of Myelodysplastic Syndromes in a Large US Cohort. American Journal of Epidemiology 169: 1492–1499. 19395696 10.1093/aje/kwp074 PMC2727203

[10] Lv L, Lin G, Gao X, Wu C, Dai J, et al (2011) Case-control study of risk factors of myelodysplastic syndromes according to World Health Organization classification in a Chinese population. American Journal of Hematology 86: 163–169. 21264898 10.1002/ajh.21941

[11] Björk J, Johansson B, Broberg K, Albin M (2009) Smoking as a risk factor for myelodysplastic syndromes and acute myeloid leukemia and its relation to cytogenetic findings: A case–control study. Leukemia Research 33: 788–791. 19019430 10.1016/j.leukres.2008.10.009

[12] Pekmezovic T, Suvajdzic Vukovic N, Kisic D, Grgurevic A, Bogdanovic A, et al (2006) A case-control study of myelodysplastic syndromes in Belgrade (Serbia Montenegro). Annals of Hematology 85: 514–519. 16691397 10.1007/s00277-006-0128-y

[13] Strom SS, Gu Y, Gruschkus SK, Pierce SA, Estey EH (2005) Risk factors of myelodysplastic syndromes: a case–control study. Leukemia 19: 1912–1918. 16167059 10.1038/sj.leu.2403945

[14] Dalamaga M, Petridou E, Cook FE, Trichopoulos D (2002) Risk factors for myelodysplastic syndromes: a case-control study in Greece. Cancer Causes Control 13: 603–608. 12296507 10.1023/a:1019573319803

[15] Nisse C, Haguenoer JM, Grandbastien B, Preudhomme C, Fontaine B, et al (2001) Occupational and environmental risk factors of the myelodysplastic syndromes in the North of France. Br J Haematol 112: 927–935. 11298587 10.1046/j.1365-2141.2001.02645.x

[16] Bjork J, Albin M, Mauritzson N, Stromberg U, Johansson B, et al (2000) Smoking and myelodysplastic syndromes. Epidemiology 11: 285–291. 10784245 10.1097/00001648-200005000-00010

[17] Nagata C, Shimizu H, Hirashima K, Kakishita E, Fujimura K, et al (1999) Hair dye use and occupational exposure to organic solvents as risk factors for myelodysplastic syndrome. Leuk Res 23: 57–62. 9933136 10.1016/s0145-2126(98)00135-0

[18] Pasqualetti P, Festuccia V, Acitelli P, Collacciani A, Giusti A, et al (1997) Tobacco smoking and risk of haematological malignancies in adults: a case-control study. Br J Haematol 97: 659–662. 9207417 10.1046/j.1365-2141.1997.942910.x

[19] Ido M, Nagata C, Kawakami N, Shimizu H, Yoshida Y, et al (1996) A case-control study of myelodysplastic syndromes among Japanese men and women. Leuk Res 20: 727–731. 8947581 10.1016/0145-2126(96)00042-2

[20] West RR, Stafford DA, Farrow A, Jacobs A (1995) Occupational and environmental exposures and myelodysplasia: a case-control study. Leuk Res 19: 127–139. 7869741 10.1016/0145-2126(94)00141-v

[21] Mele A, Szklo M, Visani G, Stazi MA, Castelli G, et al (1994) Hair dye use and other risk factors for leukemia and pre-leukemia: a case-control study. Italian Leukemia Study Group. Am J Epidemiol 139: 609–619. 8172172 10.1093/oxfordjournals.aje.a117050

[22] Crane MM, Keating MJ (1991) Exposure histories in acute nonlymphocytic leukemia patients with a prior preleukemic condition. Cancer 67: 2211–2214. 2004342 10.1002/1097-0142(19910415)67:8<2211::aid-cncr2820670835>3.0.co;2-4

[23] Pisani P, Parkin DM, Bray F, Ferlay J (1999) Estimates of the worldwide mortality from 25 cancers in 1990. Int J Cancer 83: 18–29. 10602059 10.1002/(sici)1097-0215(19991210)83:6<870::aid-ijc35>3.0.co;2-9

[24] Brugnone F, Perbellini L, Maranelli G, Romeo L, Alexopoulos C, et al (1990) [Effects of cigarette smoking on blood and alveolar air levels of benzene]. Med Lav 81: 101–106. 2250605

[25] Vigliani EC, Saita G (1964) Benzene and Leukemia. N Engl J Med 271: 872–876. 14185112 10.1056/NEJM196410222711703

[26] Aksoy M, Dincol K, Erdem S, Dincol G (1972) Acute leukemia due to chronic exposure to benzene. Am J Med 52: 160–166. 4500953 10.1016/0002-9343(72)90065-4

[27] Steensma DP (2006) Are myelodysplastic syndromes "cancer"? Unexpected adverse consequences of linguistic ambiguity. Leuk Res 30: 1227–1233. 16443272 10.1016/j.leukres.2005.12.001

[28] Sopori ML, Kozak W (1998) Immunomodulatory effects of cigarette smoke. J Neuroimmunol 83: 148–156. 9610683 10.1016/s0165-5728(97)00231-2

[29] Calderon-Ezquerro C, Sanchez-Reyes A, Sansores RH, Villalobos-Pietrini R, Amador-Munoz O, et al (2007) Cell proliferation kinetics and genotoxicity in lymphocytes of smokers living in Mexico City. Hum Exp Toxicol 26: 715–722. 17984142 10.1177/0960327107083451

[30] Mehta H, Nazzal K, Sadikot RT (2008) Cigarette smoking and innate immunity. Inflamm Res 57: 497–503. 19109742 10.1007/s00011-008-8078-6

[31] Moszczynski P, Zabinski Z, Moszczynski P Jr, Rutowski J, Slowinski S, et al (2001) Immunological findings in cigarette smokers. Toxicol Lett 118: 121–127. 11137318 10.1016/s0378-4274(00)00270-8

[32] McMillan SA, Douglas JP, Archbold GP, McCrum EE, Evans AE (1997) Effect of low to moderate levels of smoking and alcohol consumption on serum immunoglobulin concentrations. J Clin Pathol 50: 819–822. 9462262 10.1136/jcp.50.10.819 PMC500261

[33] Strom SS, Velez-Bravo V, Estey EH (2008) Epidemiology of myelodysplastic syndromes. Semin Hematol 45: 8–13. 18179964 10.1053/j.seminhematol.2007.10.003

[34] Mauritzson N, Johansson B, Rylander L, Albin M, Stromberg U, et al (2001) The prognostic impact of karyotypic subgroups in myelodysplastic syndromes is strongly modified by sex. Br J Haematol 113: 347–356. 11380398 10.1046/j.1365-2141.2001.02750.x

