# Supplementary material for: A Meta-Analysis of the Relationship between Cigarette Smoking and Incidence of Myelodysplastic Syndromes
Source: PLoS One. 2013 Jun 21;8(6):e67537. doi: 10.1371/journal.pone.0067537 (PMC3689714; doi:10.1371/journal.pone.0067537)

Figure S1. Estimates of the odds ratio of developing MDS for (A) current smokers, and (B) former smokers.


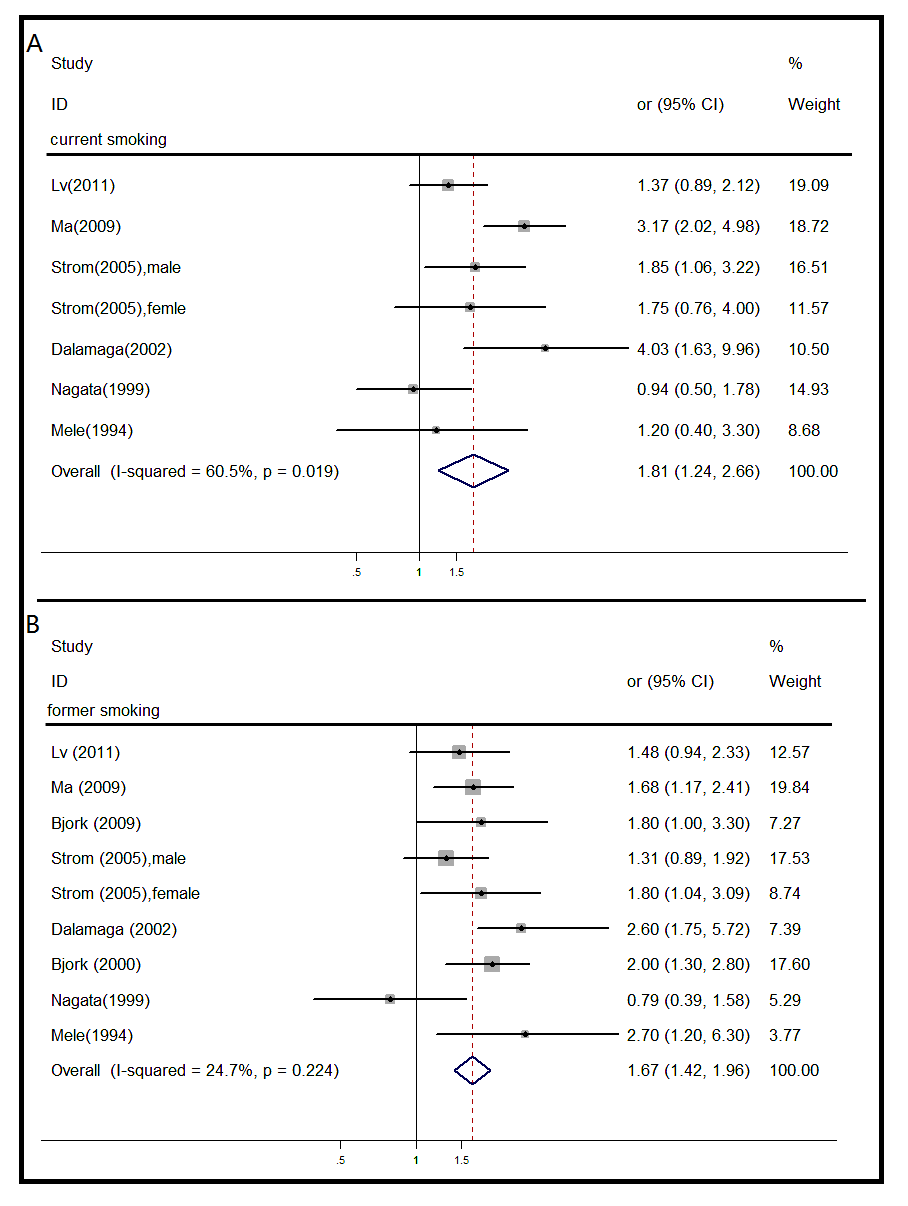

Supplement: Figure S1 — Estimates of the odds ratio of developing MDS for (A) current smokers, and (B) former smokers. (DOC) [file pone.0067537.s001.doc]
