# Supplementary material for: A Meta-Analysis of the Relationship between Cigarette Smoking and Incidence of Myelodysplastic Syndromes
Source: PLoS One. 2013 Jun 21;8(6):e67537. doi: 10.1371/journal.pone.0067537 (PMC3689714; doi:10.1371/journal.pone.0067537)

Figure S2. Forest plots showing the odds ratio of developing MDS in different subgroups: (A) geographical region; (B) gender; (C) MDS subtype.


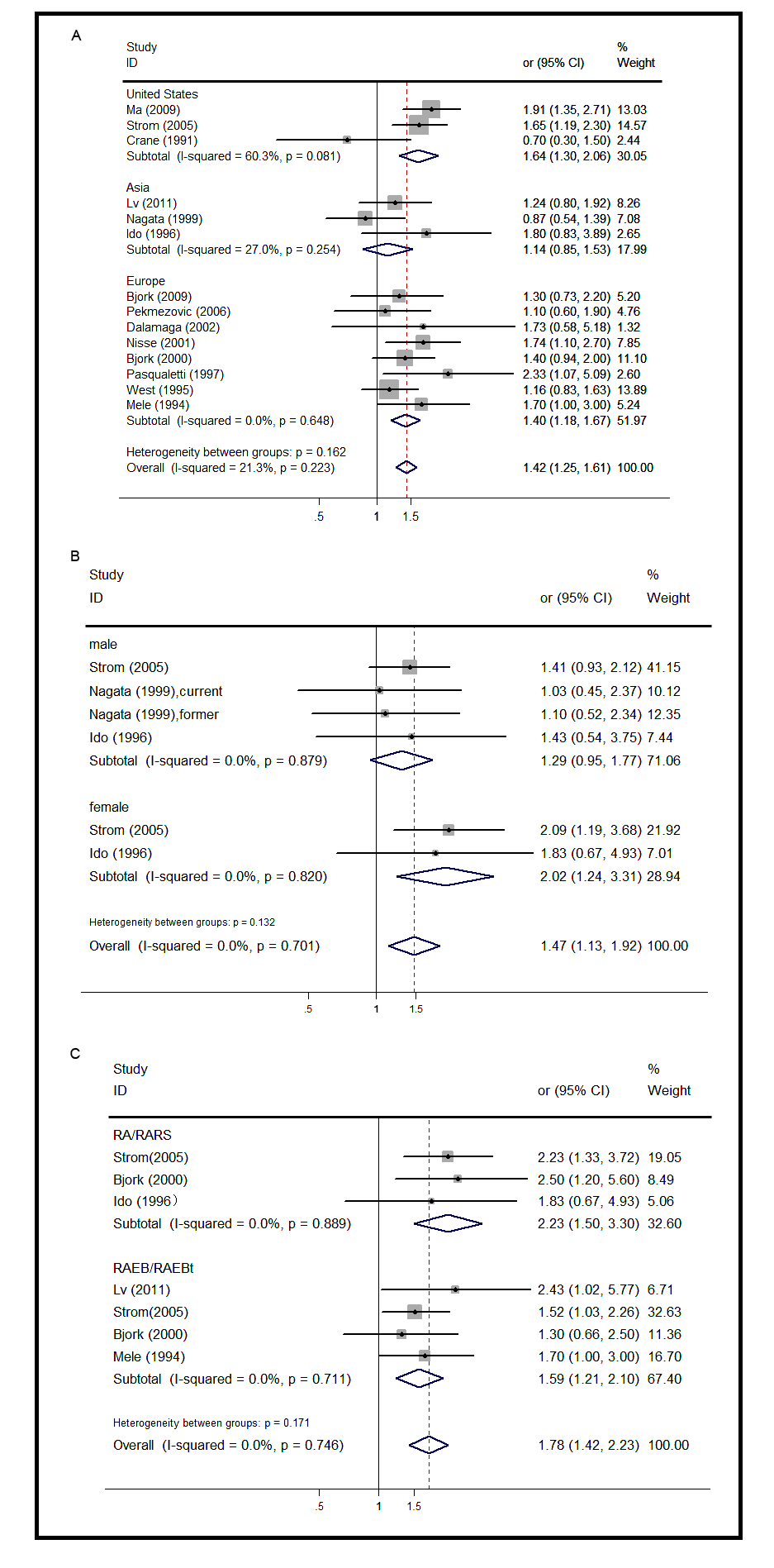

Supplement: Figure S2 — Forest plots showing the odds ratio of developing MDS in different subgroups: (A) geographical region; (B) gender; (C) MDS subtype. (DOC) [file pone.0067537.s002.doc]
